# Impact of Incentives on Physician Participation in Research Surveys: Randomized Experiment

**DOI:** 10.2196/54343

**Published:** 2024-05-14

**Authors:** Saadiya Hawa, Shalmali Bane, Kayla Kinsler, Amadeia Rector, Yashaar Chaichian, Titilola Falasinnu, Julia F Simard

**Affiliations:** 1 Graduate Medical Education Department of Internal Medicine Weiss Memorial Hospital Chicago, IL United States; 2 Department of Epidemiology and Population Health Stanford School of Medicine Stanford, CA United States; 3 Brown University School of Public Health Providence, RI United States; 4 Division of Immunology and Rheumatology Department of Medicine Stanford School of Medicine Stanford, CA United States

**Keywords:** internet survey, incentive, physician recruitment, internet surveys, online survey, online surveys, web-based survey, web-based surveys, survey, surveys, incentives, monetary incentive, monetary incentives, physician participation, physician participant, physician participants, physician, physicians, doctor participation, doctor participant, doctor participants, doctor, doctors, neurologist, neurologists

## Abstract

**Background:**

Web-based surveys can be effective data collection instruments; however, participation is notoriously low, particularly among professionals such as physicians. Few studies have explored the impact of varying amounts of monetary incentives on survey completion.

**Objective:**

This study aims to conduct a randomized study to assess how different incentive amounts influenced survey participation among neurologists in the United States.

**Methods:**

We distributed a web-based survey using standardized email text to 21,753 individuals randomly divided into 5 equal groups (≈4351 per group). In phase 1, each group was assigned to receive either nothing or a gift card for US $10, $20, $50, or $75, which was noted in the email subject and text. After 4 reminders, phase 2 began and each remaining individual was offered a US $75 gift card to complete the survey. We calculated and compared the proportions who completed the survey by phase 1 arm, both before and after the incentive change, using a chi-square test. As a secondary outcome, we also looked at survey participation as opposed to completion.

**Results:**

For the 20,820 emails delivered, 879 (4.2%) recipients completed the survey; of the 879 recipients, 622 (70.8%) were neurologists. Among the neurologists, most were male (412/622, 66.2%), White (430/622, 69.1%), non-Hispanic (592/622, 95.2%), graduates of American medical schools (465/622, 74.8%), and board certified (598/622, 96.1%). A total of 39.7% (247/622) completed their neurology residency more than 20 years ago, and 62.4% (388/622) practiced in an urban setting. For phase 1, the proportions of respondents completing the survey increased as the incentive amount increased (46/4185, 1.1%; 76/4165, 1.8%; 86/4160, 2.1%; 104/4162, 2.5%; and 119/4148, 2.9%, for US $0, $10, $20, $50, and $75, respectively; *P*<.001). In phase 2, the survey completion rate for the former US $0 arm increased to 3% (116/3928). Those originally offered US $10, $20, $50, and $75 who had not yet participated were less likely to participate compared with the former US $0 arm (116/3928, 3%; 90/3936, 2.3%; 80/3902, 2.1%; 88/3845, 2.3%; and 74/3878, 1.9%, for US $0, $10, $20, $50, and $75, respectively; *P*=.03). For our secondary outcome of survey participation, a trend similar to that of survey completion was observed in phase 1 (55/4185, 1.3%; 85/4165, 2%; 96/4160, 2.3%; 118/4162, 2.8%; and 135/4148, 3.3%, for US $0, $10, $20, $50, and $75, respectively; *P*<.001) and phase 2 (116/3928, 3%; 90/3936, 2.3%; 80/3902, 2.1%; 88/3845, 2.3%; and 86/3845, 2.2%, for US $0, $10, $20, $50, and $75, respectively; *P*=.10).

**Conclusions:**

As expected, monetary incentives can boost physician survey participation and completion, with a positive correlation between the amount offered and participation.

## Introduction

When conducting biomedical research, input from health care providers is critical in identifying barriers and facilitators to high-quality care. Such feedback occurs through multiple forums, including focus groups, interviews, and surveys. For survey research especially, participation among physicians is often low, including for web-based surveys versus postal mail [1]. While the influence of the mode of distribution, timing, and type of incentive offered has been evaluated, few studies have explored the impact of varying amounts of monetary incentives on survey completion among physicians [2-4].

We conducted a randomized study to determine to what extent the incentive amount influenced participation among neurologists participating in a case-vignette internet-based survey. For convenience, we examined neurologists as we were already conducting a larger study aimed at neurologists and could easily integrate the randomization.

## Methods

### Study Population

A mailing list of US-based specialists was obtained from SPAN Global Services or LakeMedia Group, a medical marketing company. The neurologist list (received June 1, 2022) included 22,085 email addresses. Duplicates (n=332) were removed; the remaining 21,753 individuals were randomly divided into 5 groups of ≈4351. We assigned each group to receive either US $0, $10, $20, $50, or $75 as the participation incentive.

### Survey Dissemination

For phase 1, we distributed the survey through Qualtrics (Qualtrics) using individualized email links. The surveys with the randomized incentives were sent out on August 16, 2022, followed by reminder emails on August 22, August 29, August 31, and September 9. For phase 2, we concluded the randomization of the incentives and offered each participant US $75 to complete the survey among those who had not completed the survey nor opted out. We initiated phase 2 on October 12, 2022, with reminders on October 24 and November 9. We closed the survey on November 15, 2022.

### Exposure

To provide different incentive amounts, we created 5 identical Qualtrics surveys with informed consent at the start informing participants of the randomly assigned incentive amounts: US $0, $10, $20, $50, and $75. Email text and subject lines were identical, and only the incentive amount varied. Gift card payments were managed by Tango Rewards and integrated into the survey. For the group randomized to receive no incentive (US $0), we offered a surprise US $10 gift card at the end of the survey to those who completed the survey.

### Outcomes

Qualtrics automatically reports participation as follows: email bounced, email sent, started survey, and finished survey. Our primary outcome was survey completion, defined as a “finished survey” in Qualtrics. As a secondary outcome, we considered survey participation, which included both started surveys and finished surveys.

### Statistical Analysis

We summarized self-reported characteristics among all participants who self-identified as neurologists (completed either phase 1 or 2) and then separately for phase 1 participants stratified by randomized group using descriptive statistics. Among each of the 5 randomized groups, both with (phase 1) and without (phase 2) randomized incentives, we calculated the group attrition due to bounced emails, to estimate an appropriate denominator. For phase 2, the denominator also excluded those who opted out in phase 1. For our primary outcome, we calculated the proportion who completed the survey among the respondents who presumably had the email delivered. We used chi-square tests to determine whether the proportion who completed the survey (primary outcome) and survey participation (secondary outcome) varied based on the offered incentive amount. All analyses were performed in SAS (version 9.4; SAS institute Inc).

### Ethical Considerations

The study was reviewed and approved by the Institutional Review Boards of Stanford University (approval 42909). Consent was obtained from the participants prior to beginning the survey by informing them about the format of the survey and expectations should they choose to participate. All participants were eligible to receive an incentive, including those whose initial invitation randomized them to no incentive. The amount was randomly assigned. The survey data are deidentified; however, personal information to disperse the incentives was collected and stored in a separate survey unlinked to the survey responses.

## Results

For the 20,820 emails delivered, complete responses were received from 879 (4.2%) individuals; of the 879 recipients, 622 (70.8%) were neurologists. Another 70 neurologists started the survey but did not complete it. Most participating neurologists were male (412/622, 66.2%), White (430/622, 69.1%), non-Hispanic (592/622, 95.2%), graduates of American medical schools (465/622, 74.8%), and board certified (598/622, 96.1%). Overall, 39.7% (247/622) completed their neurology residency over 20 years ago, and 62.4% (388/622) practiced in an urban setting. When restricting to phase 1 neurologist participants, we noted some modest variability in several characteristics (Table 1).

In phase 1, the proportion who completed the survey increased as the amount of incentive increased (*P*<.001; Table 2). In phase 2, an increasing proportion completed the survey in the former US $0 and $10 arms, while the US $20 arm showed no change, and the US $50 and $75 arms showed a decrease (*P*=.03; Table 2).

For our secondary outcome of survey participation, a trend similar to that of survey completion was observed in phase 1 (55/4185, 1.3%; 85/4165, 2%; 96/4160, 2.3%; 118/4162, 2.8%; and 135/4148, 3.3%, for US $0, $10, $20, $50, and $75, respectively; *P*<.001) and phase 2 (116/3928, 3%; 90/3936, 2.3%; 80/3902, 2.1%; 88/3845, 2.3%; and 86/3845, 2.2%, for US $0, $10, $20, $50, and $75, respectively; *P*=.10).

**Table 1:** Characteristics of all neurologist survey respondents (overall column) and restricted to those who completed the survey during the randomized incentive component (phase 1) stratified by the initial randomized incentive amount offered.

Characteristics			Overall (phases 1 and 2), (N=622), n (%)		Phase 1 participants by randomized incentive amount, n (%)								
					US $0 (n=32)		US $10 (n=47)		US $20 (n=59)		US $50 (n=69)		US $75 (n=84)
**Sex**													
	Male	412 (66.2)		26 (81.3)		34 (72.3)		37 (62.7)		48 (69.6)		57 (67.9)	
	Female	206 (33.1)		6 (18.8)		13 (27.7)		22 (37.3)		20 (29)		26 (31)	
	Other	4 (0.6)		0 (0)		0 (0)		0 (0)		1 (1.5)		1 (1.2)	
**Race**													
	Asian	128 (20.6)		1 (3.1)		9 (19.2)		12 (20.3)		10 (14.5)		17 (20.2)	
	Black	14 (2.3)		1 (3.1)		1 (2.1)		2 (3.4)		1 (1.5)		1 (1.2)	
	White	430 (69.1)		25 (78.1)		35 (74.5)		40 (67.8)		55 (79.7)		63 (75)	
	Other	50 (8)		5 (15.7)		2 (4.3)		5 (8.5)		3 (4.4)		3 (3.6)	
**Ethnicity**													
	Hispanic	30 (4.8)		1 (3.1)		2 (4.3)		1 (1.7)		4 (5.8)		4 (4.8)	
	Non-Hispanic	592 (95.2)		31 (96.9)		45 (95.7)		58 (98.3)		65 (94.2)		80 (95.2)	
**International medical graduate**													
	Yes	157 (25.2)		9 (28.1)		9 (19.2)		16 (27.1)		12 (17.4)		24 (28.6)	
	No	465 (74.8)		23 (71.9)		38 (80.9)		43 (72.9)		57 (82.6)		60 (71.4)	
**Completed residency**													
	>20 years ago	247 (39.7)		19 (59.4)		25 (53.2)		22 (37.3)		34 (49.3)		39 (46.4)	
	11-20 years ago	164 (26.4)		6 (18.8)		12 (25.5)		18 (30.5)		16 (23.2)		21 (25)	
	5-10 years ago	130 (20.9)		6 (18.8)		4 (8.5)		11 (18.6)		9 (13)		12 (14.3)	
	<5 years ago	67 (10.8)		1 (3.1)		4 (8.5)		6 (10.2)		6 (8.7)		11 (13.1)	
	Current resident	14 (2.3)		0 (0)		2 (4.3)		2 (3.4)		4 (5.8)		1 (1.2)	
**Board certified**													
	Yes	598 (96.1)		32 (100)		45 (95.7)		55 (93.2)		65 (94.2)		81 (96.4)	
	No	24 (3.9)		0 (0)		2 (4.3)		4 (6.8)		4 (5.8)		3 (3.6)	
**Area of practice**													
	Urban	388 (62.4)		22 (68.8)		33 (70.2)		30 (50.9)		43 (62.3)		54 (64.3)	
	Suburban	195 (31.4)		8 (25)		12 (25.5)		23 (39)		21 (30.4)		26 (31)	
	Rural	39 (6.3)		2 (6.3)		2 (4.3)		6 (10.2)		5 (7.3)		4 (4.8)	

**Table 2:** The proportion of surveys completed by neurologists during randomly assigned incentive amounts (phase 1) and after all were offered US $75 to participate (phase 2) by the initial randomized incentive amount.

Randomized survey arm	Phase 1 survey completion, n/N (%)	Phase 2 survey completion, n/N (%)
US $0	46/4185 (1.1)	116/3928 (3)
US $10	76/4165 (1.8)	90/3936 (2.3)
US $20	86/4160 (2.1)	80/3902 (2.1)
US $50	104/4162 (2.5)	88/3845 (2.3)
US $75	119/4148 (2.9)	74/3878 (1.9)

## Discussion

### Principal Findings

In this randomized study, survey completion increased as the incentive amount increased. Offering any monetary incentive was associated with survey completion, compared with offering no incentive, and those offered US $75 at outset were most likely to complete. However, participation increased disproportionately with incentive amount. Upon offering US $75 to all the groups, participation increased in the former US $0 and $10 arms, while it decreased in the other arms compared with participation during phase 1. This could occur if those likely to take the survey with any incentive offered did so during the initial distribution, which included multiple reminder emails. Another reason could be that the phase 2 incentive increase was the most substantial for the US $0 and $10 arms. Nevertheless, we saw an increasing trend across the 2 phases, with overall survey completion being 3.7% (162/4350), 3.8% (166/4351), 3.8% (166/4350), 4.4% (192/4351), and 4.4% (193/4351) for the US $0, $10, $20, $50, and $75 groups, respectively (based on phase 1 randomization). A similarly increasing trend was observed for survey participation (vs survey completion), with 3.9% (171/4350), 4% (175/4351), 4% (176/4350), 4.7% (206/4351), and 5.1% (221/4351) of individuals having started the survey for the US $0, $10, $20, $50, and $75 groups, respectively.

### Comparison With Prior Work

Researchers have studied methods of increasing physician response rates by using different strategies [2]. When paper surveys have been used, sending prenotification letters, sending stamped return envelopes, varying survey length, and packaging using hospital or medical school envelopes have been tried with some success [5-8]. Recently, a study found that the response rate for a survey was significantly higher when offered a US $50 versus US $20 check (67.8% vs 52.1%; *P*<.001) [3]. Others showed that up-front cash rewards (90/263, 34%) generate a higher response rate than an immediate check (50/255, 20%), a promised check (26/265, 10%), or a promised check with a Social Security Number requirement (20/266, 8%; *P*<.001) [9]. Our findings are consistent with previous studies of a similar nature. One recent study found that participants offered Starbucks gift cards for US $50 were more likely to respond than those offered US $25 [4]. Given the impersonal nature of an emailed survey link as in our study, lower response rates are expected compared with previous smaller studies where the physicians belonged to the same institute as the investigators. Although prepayment was shown to more than triple the participation [9] with massive distribution lists such as ours, prepayment would be cost prohibitive.

### Limitations

Our study has some limitations. The distribution list included affiliated professionals such as neurophysiologists and neurosurgeons. Some of these individuals contacted study staff for clarification about their participation, ignored the survey, or specified that they were not a neurologist in the survey (in the first question asked). This last group remained in the analysis as they were also likely to be physicians from neurology-related disciplines and our goal was to study the impact of incentive amount on survey participation among physicians. We reasoned that neurologists are a subset of physicians and, therefore, unlikely to differ from physicians as a whole. Another common limitation faced by web-based surveys distributed by email is diversion to the spam folder. To minimize this, the survey was sent from the primary investigator’s work email using a legitimate reply-to address and followed recommendations for email content and quality (eg, avoiding attachments). These limitations likely did not differ by incentive arms, given our large sample size and randomization. Unique strengths of our study were the range of incentive amounts offered; the ability to offer them immediately upon survey completion; and the ability to look at survey completion and engagement, a measure of starting the survey. Further boosting the phase 2 incentive examined the impact of visibly increasing incentives on survey completion. We found that an initial lower incentive amount followed by an increase did boost participation and may be an effective approach for studies with limited budgets. Given the large number of reminders distributed in phase 1, we do not anticipate that this was solely due to the invitation to participate with the increased amount serving as a reminder.

### Conclusions

Incentivizing physician surveys with monetary rewards can increase participation and completion. As expected, we found a positive correlation between incentive amount, participation, and survey completion. However, the increase in participation and completion observed was not proportional to the incentive amount.

## Data Availability

The data sets generated or analyzed during this study are available in a project folder on Open Science Framework. Please contact the corresponding author for necessary details to access the data.
